# Raw, Unadulterated African Honey for Ulcer Healing in Leprosy: Protocol for the Honey Experiment on Leprosy Ulcer (HELP) Randomized Controlled Trial

**DOI:** 10.2196/50970

**Published:** 2024-03-01

**Authors:** Sunday Udo, Pius Ogbu Sunday, Paul Alumbugu Tsaku, Israel Olaoluwa Oladejo, Anthony Meka, Linda Chinonso Ugwu, Motunrayo Ajisola, Joshua Akinyemi, Abiola Oladejo, Akinyinka Omigbodun, Sopna Mannan Choudhury, Jo Sartori, Onaedo Ilozumba, Sam Watson, Richard Lilford

**Affiliations:** 1 The Leprosy Mission Nigeria, Abuja, Nigeria; 2 German Leprosy and TB Relief Association/RedAid Nigeria, Enugu, Nigeria; 3 University of Ibadan, Ibadan, Nigeria; 4 University of Birmingham, Birmingham, UK

**Keywords:** leprosy, ulcers, wounds, honey, neuropathy, nerves, Africa, randomized controlled trial, RCT

## Abstract

**Background:**

Leprosy leads to nerve damage and slow-healing ulcers, which are treatable with routine therapy. There has been a recent resurgence of interest in the use of honey for the treatment of different kinds of wounds.

**Objective:**

The aim of this study, Honey Experiment on Leprosy Ulcer (HELP), is to evaluate the healing properties of raw, unadulterated African honey in comparison with normal saline dressing for the treatment leprosy ulcers.

**Methods:**

This is a multicenter, comparative, prospective, single-blinded, parallel-group, and 1:1 individually randomized controlled trial to be conducted at The Leprosy Referral Hospital, Chanchaga in Minna, Niger State, North Central Nigeria, and St. Benedict Tuberculosis and Leprosy Rehabilitation Hospital in Ogoja, Cross River State, South-South Nigeria. Raw, unadulterated honey will be used in the ulcer dressing of eligible, consenting participants in the intervention group, whereas those in the control group will be treated by dressing with normal saline. The main outcomes will be the proportion of complete healing and the rate of healing up to 84 days after randomization. Follow-up will be conducted 6 months after randomization. We aim to enroll 90‐130 participants into the study. Blinded observers will examine photographs of ulcers to determine the outcomes.

**Results:**

The recruitment of trial participants began on March 14, 2022, and has been continuing for approximately 24 months.

**Conclusions:**

Our study will provide an unbiased estimate of the effect of honey on the healing of neuropathic ulcers.

## Introduction

### Background and Rationale

Leprosy or Hansen disease is a chronic granulomatous disease caused by *Mycobacterium leprae* [1]. The disease morbidity is characterized by damage to the skin and peripheral nerves, causing neuropathy and severe disability and consequently resulting in social exclusion and stigmatization [2]. Leprosy is preventable and treatable with multidrug therapy but remains endemic in many communities of people living in poverty and with poor hygiene [2]. Nigeria reported a total of 1417 new cases of leprosy in 2020, including 87 new child cases [3], with a grade-2 disability rate of about 15% for the past 5 consecutive years [4].

A total of 30% to 50% of people infected with leprosy globally are reported to eventually have nerve damage or neuropathy [5]. Ulcers usually occur in anesthetic feet and will heal slowly with routine therapy; however, they have a tendency to recur [6].

The use of honey as a therapeutic agent in the treatment of wounds dates back to ancient times, with the earliest documented report being recorded in the *Edwin Smith Papyrus* (2600‐2200 BCE) [7]. Honey is a viscous, supersaturated solution containing sugars, water, amino acids, vitamins, minerals, enzymes, and many other substances that is derived from nectar gathered and modified by the honeybee, *Apis mellifera* [8,9]. Studies have suggested that honey promotes wound healing, stimulates tissue growth, facilitates debridement and epithelization, deodorizes, reduces edema and exudates, and possesses antimicrobial properties [7,10,11].

There has been a recent resurgence of interest in the use of honey for the treatment of different kinds of wounds, as researchers continue to search for improved, cost-effective, and readily available agents to promote wound healing [9,12]. Although there is a sizeable number of reports that show mixed levels of effectiveness in the use of honey as a topical agent for the treatment of different types of ulcers, our search of numerous databases revealed a paucity of documentation on the use of honey in the treatment of ulcers in leprosy. The effectiveness of honey to promote the healing of ulcers in general remains uncertain. A Cochrane review has reported several studies with unclear outcomes for trials with honey on venous leg ulcers, diabetic foot ulcers, and mixed chronic wounds [9]. Most of the reported studies were adjudged by the reviewers to be of low quality due to imprecision and high risk of bias. The review suggested the high risk of bias in the reports were due to nonblinding of study participants and health care professionals. Statistical heterogeneity was evident across studies [9].

The current practice for the dressing of leprosy ulcers in Nigeria is the use of normal saline [13]. Honey is acknowledged to be safe for use in wound dressings [14], with only about 5% of patients reporting pain following dressing. There is also an undocumented concern of botulism disease due to infection with *Clostridium botulinum* [15] that is associated with the use of honey, as it is considered a suitable environment for the anaerobic bacteria to thrive. This study will evaluate the effectiveness of honey in the treatment of ulcers in leprosy in comparison with normal saline dressing.

### Objectives

The aim of this study, Honey Experiment on Leprosy Ulcer (HELP), is to evaluate the healing properties of raw, unadulterated African honey in comparison with normal saline dressing for the treatment of leprosy ulcers.

The study objectives are as follows:

1. Recruit 90‐130 eligible, consenting people within 12 months.
2. Randomize the participants to receive ulcer dressing with either honey (intervention group) or normal saline (control group) twice a week.
3. Observe the rate of healing based on 2 observations per week (cm^2^ per unit time, until either the ulcer has healed or 84 d have elapsed since randomization).
4. Observe the time to healing, defined as complete re-epithelization (up to a maximum of 84 d).
5. Monitor the rate of activity of study participants with pedometers.
6. Monitor the recurrence of treated ulcers or appearance of new ulcers and any anatomical changes in the limb at 6-month follow-up after randomization.

### Trial Design

**Figure 1.**
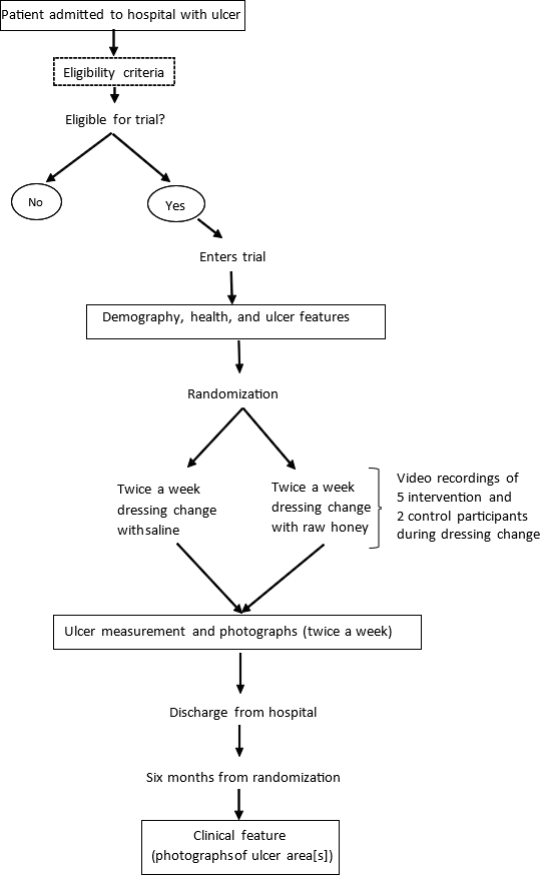
Honey Experiment on Leprosy Ulcer (HELP) study pathway.

The study is a multicenter, prospective, single-blinded, parallel group, and 1:1 individually randomized controlled trial. The study duration is 48 months (maximum), with recruitment starting at month 5 and postdischarge follow-up starting at month 11. We aim to enroll between 90 and 130 participants over a 24-month recruitment period. The study pathway is shown in Figure 1.

## Methods

### Study Setting

The study centers are The Leprosy Referral Hospital, Chanchaga in Minna, Niger State, North Central Nigeria, and St. Benedict Tuberculosis and Rehabilitation Hospital in Ogoja, Cross River State, South-South Nigeria. The main study will be preceded by prestudy due diligence.

The Leprosy Referral Hospital, Chanchaga in Minna, Nigeria, is a specialist hospital operated by the government of Niger State in collaboration with The Leprosy Mission (TLM) Nigeria. The hospital was established in 1940 and has 2 wards (eye and physiotherapy units), a theater, a laboratory, and a dispensary. The hospital is supported by an orthopedic workshop that is built and managed by TLM Nigeria, which produces assistive devices including wheelchairs, crutches, protective sandals, and artificial legs.

St. Benedict Tuberculosis and Rehabilitation Hospital is a tuberculosis and leprosy referral hospital located in Ogoja, a town in the northern part of Cross River State of Nigeria. It is owned by the Catholic Diocese of Ogoja, Cross River State, Nigeria. It provides these services in collaboration with the National Tuberculosis and Leprosy Control Programme and with the technical assistance of German Leprosy and TB Relief Association. The center has a community outreach program, which covers 31 communities in the Bekwarra, Ogoja, and Yala local government areas of Cross River State.

### Ethical Considerations

The trial will be conducted in full conformance with the principles of the Declaration of Helsinki and Good Clinical Practice Guidelines. It will also comply with all applicable UK legislation and standard operating procedures for University of Birmingham–sponsored studies. Ethics approval has been granted in Nigeria for the study by the National Health Research Ethics Committee (approval FHREC/2022/01/09/04-02-22) and Niger State Ministry of Health (reference STA/495/Vol/199).

### Eligibility Criteria

The local, medically qualified researchers employed on the grant will screen all ulcer admissions. The researchers will complete web-based eligibility forms for each patient with an ulcer. Consent will be sought by the research fellow from consecutive patients aged ≥18 years with a foot or leg ulcer between 2 and 20 cm^2^ and not requiring surgery (eg, skin graft). The inclusion criteria are as follows:

1. Patients with a chronic foot ulcer of at least 6-week duration due to leprosy neuropathy.
2. Patients ≥18 years of age.
3. Ulcer with a surface area between 2 and 20 cm^2^ inclusive.
4. Ulcer is clean, dry, and free from infection.
5. Patient can provide informed consent.

The exclusion criteria are as follows:

1. Any significant medical condition, laboratory abnormality, or psychiatric illness that would prevent the participants from participating in the study.
2. Ulcers with a surface area <2 or >20 cm^2^.
3. Patient requires a skin graft.
4. Any condition that confounds the ability to interpret data from the study (ie, patients with HIV or tuberculosis under active treatment).
5. Any wound that has clinical microbial infections.
6. Diabetes or diabetic ulcer.
7. A patient has returned to the hospital having already taken part in the trial.

The wound dressing protocol for the HELP study is shown in Multimedia Appendix 1.

In the uncommon scenario where a participant has more than 1 foot ulcer, the largest ulcer will be selected as the index ulcer before randomization, and all ulcers will receive the same treatment (eg, if the participant is in the intervention group, all ulcers—not just the 1 being monitored for the trial—will be treated with honey). Observations will be made on all ulcers, but only the largest ulcer will be used in the primary analysis (see the *Discussion* section). Eligible patients will be offered entry in the trial at the point where their senior clinician judges them to be suitable for the honey treatment. This point arises once the lesion has been cleared of any debris or infection, typically by 7‐10 days after beginning treatment with or without antibiotic or debridement.

Swabs of the ulcer area will be taken to assess the wound for bacterial infections prior to the recruitment of participants into the study.

### Informed Consent Collection

Researchers at the study center have been trained on Good Clinical Practice. They will be responsible for the screening of eligible participants and taking of consent. The participant information sheet has been translated into the local language (Hausa). All relevant information for the research participants on the study are contained in the participant information sheet. The information sheet will be given to the participants a day before enrollment into the study. The content of the information sheet will be explained to them verbally, and they will be encouraged to ask questions should they need more clarification. Written informed consent will be sought from the eligible participants after the study has been explained to them and they have taken time to decide to enroll into the study. The consent form will be signed or thumb- or fingerprinted by the participants before enrollment.

### Additional Consent Provisions for the Collection and Use of Participant Data and Biological Specimens

The participant information sheet contains all information about the data to be collected and how the data will be stored and used. Biological specimens such as a swab of the ulcer surface will be obtained for the purpose of screening for bacterial infections prior to the recruitment of a participant into the study. Participants who refuse to give consent will continue to receive normal care from the hospital, but they will not be part of the study.

Participants can decide to withdraw their consent at any time during the study. Such participants will be given a form to complete, stating their reason for withdrawal. A participant may either withdraw fully or they may withdraw from treatment but consent to ongoing data collection.

Once a person consented to participate in the trial, baseline data will be collected. This will precede randomization.

Data will be collected directly onto electronic tablets, and the computer program (Research Electronic Data Capture [REDCap]; Vanderbilt University) will check the range of the information. Photographs of the ulcers will be captured using the tablet device. Each participant will be allocated a unique trial number, which will be included in all data entry forms and linked to the photographs. The level of activity (step count) will be recorded at each dressing change across both intervention and control groups until 84 days or discharge, whichever comes first. This date will enable us to check for postrandomization (“performance”) bias.

### Interventions

#### Explanation for the Choice of Comparators

The participants in the control group will receive the usual care of twice weekly normal saline dressings only. Normal saline dressing is the standard ulcer dressing method currently in use in the hospitals where the trial is taking place.

#### Intervention Description

The intervention for this study is raw, unadulterated honey obtained from local bee farmers in North Central Nigeria. The honey samples have been examined at The National Institute for Pharmaceutical Research and Development, Abuja, and confirmed to be free from microbial contaminants. The honey is stored in airtight plastic containers at room temperature and away from direct sunlight. The honey will be applied topically to the wound during dressing under strict hygienic conditions by using sterilized equipment.

The treatment will be applied at twice weekly dressing changes by local trained nurses or paramedics. These dressing changes are part of routine care and will thus be applied to the intervention and control groups. Dressing changes may take slightly longer for participants in the intervention group, but pain is unlikely as the affected limb has a loss of sensation. Participants in both groups have twice weekly dressing changes during their hospital stay until the ulcers are healed. Any missed sessions will be noted, but this will not be treated as a protocol deviation.

#### Interval Pilot to Monitor the Blinding of Observations

We are concerned that honey residue may be discernible by the observers of the ulcer outcome. We will therefore conduct an interval pilot to examine this issue. To ensure proper blinding of the trial, images of the ulcers from the second dressing change (following the honey or saline application at the first dressing change) for the first 10 enrolled participants will be sent to 3 independent assessors in Nepal. The assessors will look out for traces of honey residues that might interfere with blind assessment during the study. If the assessors are able to distinguish between cases treated with honey and controls, then we will consider alternating honey and saline dressings and making weekly observations rather than twice weekly observations.

We will also make a video recording of the first dressing change for the first 5 intervention and 2 control participants and send it to the 3 independent assessors. An uninterrupted video recording will be made from before the dressing is removed until after it has been replaced.

### Relevant Concomitant Care Permitted or Prohibited During the Trial

In line with standard practice, participants will be discouraged from bearing weight on the ulcer site, since weight bearing and the patients’ level of activity might affect the ulcer’s rate of healing. Participants will be asked to wear pedometers on the nonaffected foot, and the level of activity will be recorded on the tablet at each dressing change from the first dressing change until 84 days from randomization or discharge, whichever comes first.

### Provisions for Posttrial Care

The date of discharge will be noted, along with participant contact details, addresses, and contact details for at least 1 family member. The participants will be routinely contacted 6 months after randomization. The treated ulcer area will be examined and photographed. The researcher will take photographs of the ulcer area (healed in the majority of cases). The dates covering any readmissions to any hospital for the treatment of the “trial ulcer” will be recorded.

### Outcomes

We define 2 main outcomes relating to ulcer healing. The main outcomes will be (1) rate of healing based on 2 observations per week (until healed or 84 d from randomization) and (2) time to healing defined as complete re-epithelization (up to 84 d). Secondary outcomes will be (1) the recurrence of treated ulcer and (2) appearance of a new ulcer. Long-term outcomes will be measured at the follow-up 6 months after randomization, which will be (1) the number of days hospitalized prior to discharge and in total (to include any readmission related to leprosy ulcers) by 6 months and (2) the number of visits to any health care facility from discharge to the end of follow-up at 6 months.

### Participant Timeline

**Figure 2.**
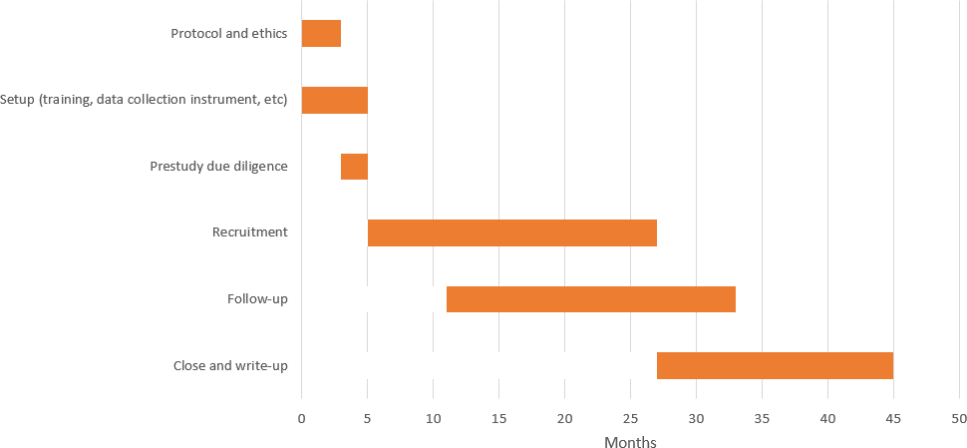
Timeline (in months) for the Honey Experiment on Leprosy Ulcer (HELP) trial (based on the most pessimistic estimate).

Participants’ timeline through the trial is outlined in Figure 2.

### Sample Size

Recruitment will continue for 24 months. We expect to enroll between 90 and 130 participants. Sample size was based on the 2 clinical outcomes: the rate of healing and time to complete re-epithelialization. We expect at least 70% of ulcers to heal within 84 days with standard care (according to a recent study of neuropathic ulcers, with over half due to leprosy [16]). If we assume that the intervention will increase this proportion to 90% and hazards are constant and proportional (so that the hazard ratio is 1.91 for discharge), for a 2-sided test of the hazard ratio with a type I error of 5%, a statistical power of 80%, and a 1:1 allocation ratio, 47 individuals are required in each group. With 130 participants, this allows for a dropout rate of up to 40% to achieve a power of 80%. At the most pessimistic sample size of 90, with a dropout rate of 40%, our minimum detectable effect size (ie, the effect size that achieves a power of 80% with 33 patients per arm) is a hazard ratio of 2.15, or 92.5% of patients in the treatment group being discharged by the end of the trial period. At the most optimistic sample size of 130 with no dropout, our minimum detectable effect size is a hazard ratio of 1.74, or 87.3% of patients being discharged in the treatment group. All calculations are based on a log-rank test.

We took a conservative approach and based the sample size calculation on the healing outcome and model with lower efficiency to ensure an adequate sample size for all main outcomes, since our inferential approach is not based on statistical significance but on a consideration of effect sizes and a comparison and triangulation of the totality of evidence. We are not particularly concerned with being “overpowered” or with a problem of multiple comparisons; instead, we will focus on ensuring a sufficient sample size to estimate clinical effectiveness to a reasonable degree of precision [17,18].

### Recruitment

Eligible individuals are identified by the on-site clinical research team and are invited to take part in the study.

### Assignment of Interventions: Allocation

#### Sequence Generation

Participants will be enrolled sequentially, stratified by ulcer size (above or below 10 cm^2^) and randomly allocated (1:1) to undergo honey treatment or usual care with normal saline using a “digital sealed envelope” method [19].

#### Concealment Mechanism

An allocation table will be generated remotely at the trials office at The University of Ibadan. A permuted block randomization method will be used to generate the randomization sequence within each stratum. Randomly selecting blocks of size 2, 4, 6, or 8 will be generated in order to maintain balance between the numbers allocated to each of the 2 groups and to ensure allocation concealment. The generated table will be uploaded into the REDCap database platform to be used for participant enrollment. Access to the allocation table will be restricted. Trial staff in Nigeria will not have access to the allocation table. When a participant’s details are submitted, the trial arm and a unique study number will be assigned and revealed to the local clinician so that the randomized group that the participant is assigned to cannot be altered.

#### Implementation

A trial statistician at The University of Ibadan will generate the allocation sequence while researchers and clinical staff on-site will enroll and assign participants to the respective control or intervention groups.

### Assignment of Interventions: Blinding

#### Researcher Blinding

Only the overall on-site research supervisor, the database managers at the University of Birmingham and the University of Ibadan Trials Unit, the clinical staff carrying out dressing changes in the room designated for this purpose, and the participants themselves will be aware of the participants’ randomly assigned group. Ward staff will not be informed. The assessors in Nepal will be blinded from the treatment provided for the randomly assigned groups.

#### Procedure for Unblinding if Needed

There is no provision for the unblinding of trial participants during the trial.

### Data Collection and Management

#### Plans for the Assessment and Collection of Outcomes

Standardized photographs [20] will be taken twice weekly of the dressing changes during the in-patient stay for participants in both intervention and control groups. Any residual honey will be gently removed with a wet swab. Photographs will be taken using the built-in camera in the tablet devices (Samsung Galaxy Tab S7, 13Mp). The photographs will be taken perpendicular to the ulcer. For calibration purposes, a 3-cm clean paper ruler with the date and participant’s trial identification number will be placed in the photograph frame above or below the ulcer but at the level of the skin. The photographs will then be uploaded onto the REDCap database platform and accessed by database managers at the University of Birmingham. The ulcer photos will then be randomized and sent to the blinded assessors in Nepal for the measurement of ulcer dimensions. The photographs will be evaluated digitally by the designated observer in Nepal using the PictZar Digital Planimetry Software with the electronic PUSH Tool (National Pressure Injury Advisory Panel [21]). The observer will delineate an area of interest by manually “painting” the ulcer area with color using a computer mouse. The software will then calculate the ulcer dimensions based on this profile.

The assessors will be blinded to the treatment allocated to the participant. The assessors are all experienced in leprosy, ulcer care, and the measurement of ulcers using the PictZar software. All photographs from a given participant will be assigned to the observers at random. To ensure that the measurements are not all relegated to the end of the study, they will be made in batches of 10 participants—when they reach the completion of their treatments. A proportion (20/100, 20%) of all ulcer photographs will be measured by 2 assessors to estimate interrater reliability. These photographs will be selected at random.

Treatment will stop when the local clinician determines that complete epithelization has taken place. Photographs will be taken at this point and at the follow-up visit.

#### Plans to Promote Participant Retention and Complete Follow-Up

TLM Nigeria has a meal subsidy program for all in-patients at The Leprosy Referral Hospital, Chanchaga. The meal subsidy program will continue through the period of this study. It is expected that the meal subsidy program will be sufficient to keep the patient at the hospital from the period of admission up to the time of discharge. Phone contacts of participants or their close relatives will be collected and used for postdischarge follow-up.

#### Data Management

All data generated from this study will be classified according to the University of Birmingham Information Security Framework. All data will be collected and stored electronically to reduce data entry errors, such as contradicting answers. Data will be reported on an electronic case report form, and all local staff will be trained to collect data directly onto electronic tablets. Data will be acquired and stored on the REDCap database platform, with access restricted by passwords at both the University of Ibadan and the local site in Nigeria. Each participant will be allocated a unique study number when they agree to participate (and before randomization), which will be used on all documents. A master list linking a trial participant number to their identity (name) will be retained by the hospital securely in a locked filing cabinet. This is necessary so that the notes of any person who withdraws consent for data storage can be removed. The trial participant master list will be stored separately from patient lists and the trial data.

#### Confidentiality

Participant confidentiality will be ensured throughout the study and no participant-identifying information will be released to anyone outside the project. Confidentiality will be secured through several mechanisms. Each participant will be assigned a study subject ID, which will be used on all study forms. Any study forms and paper records that contain personal identifier information (eg, address lists and phone lists) will be kept secured and locked at the trial sites. Access to all participant data and information at the sites will be restricted to authorized personnel.

Participants will not be identified by name in any reports or publications, nor will the data be presented in such a way that the identity of individual participants could be inferred. Analysis files created for further study by the scientific community will have no participant identifiers. The trial data will be kept under lock for up to 10 years, after which they will be destroyed. Only authorized persons will be granted access to the trial data.

#### Plans for Collection, Laboratory Evaluation, and Storage of Biological Specimens for Genetic or Molecular Analysis in This Trial or for Future Use

At the pre-enrollment stage, we will collect a swab of the ulcer surface to examine the wound for bacterial infections. Afterward, we will not collect or retain biological specimens for any future use in this trial.

### Statistical Methods

#### Statistical Methods for Primary and Secondary Outcomes

Time to healing will be analyzed using a Cox proportional hazards model with and without adjustment for baseline characteristics (trial ulcer area and participants’ age), allowing for right censoring. For the rate of healing, we will define the outcome ulcer size in cm^2^ at each time point and include in the model time since admission, treatment status, and their interaction. We will analyze this model using a linear mixed-effects model with participant-level random effects and both with and without adjustment for participant characteristics. Given there are multiple clinical outcomes (2 outcomes, with and without adjustment), we will adjust reported *P* values for multiple testing using a step-down method, which provides an efficient means of controlling the family-wise error rate [22]. We will derive the approximate distributions of the test statistics to perform the step-down procedure using a permutation test approach, by simulating 10,000 rerandomizations of the individuals [23].

#### Interim Analyses

An interim analysis will be conducted when at least 49 (three-eighths [37.5%] of the 130 target) participants have been followed up for of 84 days or discharged, whichever comes first. The rationale for this analysis is the detection of a “penicillin-like” benefit or statistically significant negative effect of the treatment on either primary outcome. The statistical methods will be as specified above. A statistical threshold of 0.01, one-sided (0.02, two-sided), will be used for either primary outcome. If either threshold is exceeded, or if the Independent Data Monitoring Committee (IDMC) has other concerns, then the Trial Steering Committee (TSC) will be advised accordingly. If the IDMC wishes to meet again before trial conclusion, they will be able to do so. Only the trial statistician and the IDMC will see the “unblinded” data, unless the TSC is informed.

#### Methods for Additional Analyses

We will also compare average daily step count between treatment and control groups as a simple difference in means (1-tailed *t* test). Since 1 group may stay longer in hospital than the other and since there may be an interaction between the rate of healing and step count, we will compare step counts over preset periods at 7, 14, and 42 days.

#### Analytical Methods to Handle Protocol Nonadherence and Statistical Methods to Handle Missing Data

In the unlikely event of missing data by any reason, such as the withdrawal of participant before the completion of treatment, and that the rate differs between groups, a sensitivity analysis will be carried out. The pattern and rates of missing data that are particularly related to the end points will be explored. Strategies such as multiple imputation within the sensitivity analysis will be explored instead of imputing missing values.

#### Plans to Give Access to the Full Protocol, Participant-Level Data, and Statistical Code

The full protocol, nonidentifiable participant-level data, and statistical code may be available for sharing once the trial has ended. All requests will be approved by Chief Investigator RL.

### Oversight and Monitoring

#### Composition of the Coordinating Center and TSC

TLM Nigeria is the study sponsor and will oversee the study process in Nigeria. The University of Ibadan will provide data management services and perform quality checks, whereas the University of Birmingham will house the data securely and perform quality checks.

The Trial Management Group (TMG) includes individuals at the University of Birmingham; The Leprosy Referral Hospital, Chanchaga; TLM Nigeria; the German Leprosy and TB Relief Association/RedAid Nigeria; and the University of Ibadan Clinical Trials Unit who are responsible for the day-to-day management of the trial. The TMG will meet monthly by teleconference, but this may be more frequent if deemed necessary by the members.

The TSC will provide overall supervision of the trial and ensure that it is being conducted in accordance with the principles of Good Clinical Practice and other relevant regulations. The TSC will have oversight of the trial. It will send its report to the overall Research and Innovation for Global Health Transformation program steering committee, but the TSC will have the final say with the running of the trial itself. The TSC will meet either face-to-face or via teleconferencing. Meetings will be scheduled for before the enrollment of participants begins, following each meeting of the IDMC, and then during the analysis phase or more often if required.

#### The IDMC’s Composition, Role, and Reporting Structure

The IDMC consists of individuals who have no conflict of interest within the study. Safety and efficacy data will be supplied, in strict confidence, for review by the IDMC after the 49th participant has been followed up for of 84 days or discharged, whichever comes first. The IDMC will be asked to give advice on whether the accumulated data from the trial, together with the results from other relevant research, justifies the continuing recruitment of further participants. The IDMC will meet either face-to-face or by teleconferencing. An emergency meeting may also be convened if a safety issue is identified. The IDMC will escalate any issues and recommendations to the TSC, who will then make decisions based on these recommendations.

### Adverse Event Reporting and Harms

#### Overview

The principal investigator in Nigeria will be responsible for recording all adverse events (AEs) and reporting any serious AEs to the University of Birmingham and the University of Ibadan within 24 hours of the research staff becoming aware of the event. A serious AE form will be available on the tablets used to collect data, and we will maintain a database of all safety events or AEs. The forms will be reviewed by the TMG, which meets monthly, and by the project manager, if required. The TSG will periodically review all safety data and liaise with the IDMC regarding any safety issues.

Any deaths will be reported to the sponsor, irrespective of whether the death is related to the disease progression, the intervention, or an unrelated event. Only deaths that could plausibly be caused by the intervention will be reported to the sponsor immediately.

#### Frequency and Plans for Auditing Trial Conduct

The trial is audited and monitored by the sponsor, TLM Nigeria.

#### Plans for Communicating Important Protocol Amendments to Relevant Parties

Any protocol amendment will be reported to the TMG to approve the change. The amendment will be sent to the sponsor to confirm substantiality and then to the National Health Research Ethics Committee for approval.

### Dissemination Plans

The results of the trial will first be reported to trial collaborators. The main report will be drafted by the trial coordinating team, and the final version will be agreed by the TSC before submission for publication, on behalf of the collaboration.

We will present our work at conferences such as the annual conference of the Neglected Tropical Disease NGO Network, of which TLM is a member; the Health Systems Global Conference; and the International Leprosy Congress in 2024. Other dissemination plans include bite-sized research reports in lay format, public announcements in communities in low- and middle- income countries, policy briefings, print and web-based media, the chief investigator’s news blog (680+ subscribers), and institutional and professional social media accounts and websites.

## Results

The recruitment of trial participants began on March 14, 2022, and has been continuing for approximately 24 months.

## Discussion

This study protocol describes a clinical trial with honey as an intervention for the dressing of leprosy ulcers. It is a single-center, comparative, prospective, single-blinded, parallel-group, and blocked-stratified randomized controlled trial.

Leprosy is regarded as a neglected tropical disease because of its near absence from the global health agenda [24]; as such, very little attention has been given to the treatment of ulcers in leprosy in spite of its debilitating nature and the social stigma it attracts. Methods such as laser therapy [25], zinc tape [26], pentoxifylline injection [27], amniotic membrane gel [28], phenytoin suspension [29], and leukocyte- and platelet-rich fibrin [3] have been tested on leprosy foot ulcers as the search for the most suitable treatment continues. Normal saline has been the most common comparator in most of the studies on ulcers in leprosy [30]. Among the challenge with the normal saline dressing are the slow rate of healing, the possibility of ulcers getting infected with pathogens [31], and the likelihood of the recurrence of treated ulcers. A Cochrane review [30] noted that previously published evidence is limited, due to a high or unclear risk of bias (selection, performance, detection, or attrition), imprecision due to the small number of participants, indirectness due to poor outcome measures, and inapplicable interventions.

Honey is considered as a cheaper and more readily accessible alternative treatment for many types of wounds. Although honey has been known for centuries to promote wound healing, there are only a few controlled clinical trials that assess its efficacy. While honey is noted to have wound healing properties, including debridement, deodorization, and antimicrobial properties [14], its use may result in painful sensation in about 5% of persons, although this is unlikely to occur in leprosy and diabetes [15]. The hypothetical risk of botulism will be mitigated by regularly screening the honey sample. Detailed procedures for the study have been described in this protocol. We expect that the outcome of this study will provide valuable evidence on the use of honey for the treatment of ulcers in leprosy.

## Supplementary material

10.2196/50970 Multimedia Appendix 1 Wound dressing protocol.

## Abbreviations

AE: adverse event
HELP: Honey Experiment on Leprosy Ulcer
IDMC: Independent Data Monitoring Committee
REDCap: Research Electronic Data Capture
TLM: The Leprosy Mission
TMG: Trial Management Group
TSC: Trial Steering Committee
