# Supplementary material for: Raw, Unadulterated African Honey for Ulcer Healing in Leprosy: Protocol for the Honey Experiment on Leprosy Ulcer (HELP) Randomized Controlled Trial
Source: JMIRx Med. 2024 Mar 1;5:e50970. doi: 10.2196/50970 (PMC11024902; doi:10.2196/50970)
Supplement: Multimedia Appendix 1 [file xmed-v5-e50970-s001.docx]

Honey Experiment on LeProsy Ulcer (HELP): A Randomised Control Trial of Raw, Unadulterated African Honey for Ulcer Healing in Leprosy

**Wound Dressing Protocol**

**Funding body:** UK National Institute for Health Research (NIHR) Research and Innovation for Global Health Transformation (RIGHT) Programme

**Ethics approval date:** 6^th^ October 2021

**Version number of approved protocol:** 0.8

**Sponsor:** The Leprosy Mission Nigeria

**Clinical centre:** The Leprosy Referral Hospital, Chanchaga, Niger state

**Chief Investigator (Birmingham):** Professor Richard Lilford

**Principal Investigator (Nigeria):** Dr Sunday Udo

**Wound dressing protocol for HELP study**

If Patients fulfil the inclusion criteria and offer to enrol them in the study.

**Screening and consent taking:-**

1. Participant admitted to hospital.

2. Check the Patients’ vitals. Note down all parameters.

3. Send ulcer swab for culture to lab and check for any microbial growth and their antibiotic sensitivity. Irrigate wound with normal saline before taking swab.

4. After receiving the reports, screen the participants for eligibility using REDCap and record size of ulcer- length, breadth, depth and area in cm/cm^2^ using measurement scale.

5. If participant is eligible and willing to take part in the trial, take informed consent.

6. The patients should have the details of the procedure explained to them on the first day and written consent should be provided the following day with signature or fingerprints.

7. Collect baseline data, following consent, before randomization

**Wound Preparation for both Control and Intervention Groups:**

1. Fill out the participant e-CRF (case report form) into REDCap.

2. Record time as hour: minutes so that we can calculate the time taken for dressing change. It should be the duration of time from opening the dressing until closing the wound with bandaging.

3. Clean the wound with normal saline through irrigation to remove all exudates.

4. Wound debridement - remove the devitalized tissue and fibrin membrane surrounding the ulcer by careful debridement (marginal resurfacing) of the wound. Clean the wound with normal saline. Ulcer debridement is not recommended at every dressing change.

5. Take photographs perpendicular to wound with a labelled (Participant Identification Number and Date) 3cm ruler at the level of the skin and calculate measurements using PUSH tool, and record these locally. Record the wound- length, breadth, depth and surface area in cm/cm^2^. Measurements should not be recorded in REDCap software by the Nigeria team, as wound assessment will be performed by a team from TLM Nepal blind to intervention status. Where an index ulcer heals into multiple smaller ulcers, the surface area of all ulcers within the original index ulcer site will be recorded in cm^2^.

**Randomization:**

All participants will be allocated into 2 groups according to computer generated randomisation schedule:

1. Twice a week dressing with normal saline (Control group)

2. Twice a week dressing with Honey (Intervention Group)

**Honey Application to the Wound for Intervention Group:**

1. Filter the honey into sterile container using sterile polypropylene 0.5 micron filter cloth.

2. Prepare clean dry gauze and soak with the filtered honey. Apply the honey-soaked gauze directly on the wound.

3. Cover the wound with sterile Vaseline gauze and apply sterile dry gauze on it, close wound with bandaging. Apply liquid paraffin to the surroundings of the wound if the foot skin is dry. Convince the participants to avoid weight bearing at the wound site. There is no need of dressing every day. Record the total time taken for dressing change as hour: minutes.

4. Open the wound after 3-4 days as per twice a week dressing schedule. Clean the wound with normal saline, dry it with gauge, and do not rub the wound. Take photographs, note down the measurement of wound size and depth.

**Normal Saline Wound dressing for Control Group:**

Wound preparation procedure is same as mentioned above.

1. Perform irrigation with 50-100 ml Normal saline depending on the size of the ulcer. Apply normal saline gauge over the wound area and cover with sterile Vaseline gauze. Apply sterile dry gauze over it and close wound with bandaging. Apply liquid paraffin to the surroundings of the wound if the foot skin is dry. Convince the participants to avoid weight bearing at the wound site. There is no need of dressing every day. Record the total time taken for dressing change as hour: minutes.

2. Open the wound after 3-4 days as per twice a week dressing schedule. Repeat the Normal saline dressing as mentioned above. Do not rub the wound surface. Take photographs, note down the measurement of wound size and depth.

**Implement following to all participants in both Intervention and Control groups-**

Provide a glass of energy drink to participants every time after dressing change.

Add Iron tablets (200 mg BD), folic acid (5 mg OD), Vitamin C (500 mg BD) and Multivitamins (1 tab BD) to the participant’s medication list during admission period.

**Post wound healing care and Follow up:**

1. If the wound is completely epithelialized before 70 days after enrolment, check for the consistency of tissue at wound site. Take a photo with PUSH tool tablet camera of whole foot. Apply Vaseline gauge at healed wound site and liquid paraffin around it, then apply Plaster of Paris (PoP) cast with window at healed wound site for 2-4 weeks until the healed ulcer site will have matured tissue.

Participants will be discharged from hospital after POP application. They can be transferred to Self-Care Unit until the PoP will be removed out or can be sent to their home. The POP should be removed after 2-4 weeks.

2. After removal of PoP, train the participants for self-care and prevention of disability.

3. Consult footwear staff to design protective footwear.

4. Discharge the participants from Self-Care Unit and ask them to come at six months from randomisation for follow up evaluation.

5. At follow up check condition of foot and record any recurrence or new ulcer at same foot.

6. Take photographs with PUSH tool at follow-ups.

7. If any complications or adverse events are seen during admission period, please inform the Principal Investigator or RIGHT Research Staff.

## **Points of contact**

| **General Protocol Questions** | *Chief Investigator:- Professor Richard Lilford* [r.j.lilford@bham.ac.uk](mailto:r.j.lilford@bham.ac.uk)  *Principal Investigator:- Dr. Sunday Udo* [*sundayudotlmn@gmail.com*](mailto:sundayudotlmn@gmail.com)  *Co-investigator:- Pius Sunday Ogbu* [*piuss@tlmnigeria.org*](mailto:piuss@tlmnigeria.org)  *Co-investigator:- Dr Paul Tsaku* [*tsakup@tlmnigeria.org*](mailto:tsakup@tlmnigeria.org) |
| --- | --- |
| **Urgent Clinical Questions** | *Co-investigator:- Dr Paul Tsaku* [*tsakup@tlmnigeria.org*](mailto:tsakup@tlmnigeria.org)  *Isreal Oladejo* [*physioeasy1@gmail.com*](mailto:physioeasy1@gmail.com) |
| **Safety and Protocol Violations/Waivers** | *Co-investigator:- Dr Paul Tsaku* [*tsakup@tlmnigeria.org*](mailto:tsakup@tlmnigeria.org) |
| **Statistician** | *Dr. Joshua Akinyemi* [odunjoshua@yahoo.com](mailto:odunjoshua@yahoo.com) |
| **Clinical Trials Unit Senior Project Manager** | *Dr. Motunrayo Ajisola* [ayobolamotunrayo@gmail.com](mailto:ayobolamotunrayo@gmail.com) |
| **Study Manager** | *Sopna Choudhury S.M.Choudhury.1@bham.ac.uk* |
| **General Study Questions** | *Dr Paul Tsaku* [*tsakup@tlmnigeria.org*](mailto:tsakup@tlmnigeria.org) |
